# Poor mental health of livestock farmers in Africa: a mixed methods case study from Ghana

**DOI:** 10.1186/s12889-020-08949-2

**Published:** 2020-06-01

**Authors:** Francis Sena Nuvey, Katharina Kreppel, Priscilla Awo Nortey, Adolphina Addo-Lartey, Bismark Sarfo, Gilbert Fokou, Donne Kofi Ameme, Ernest Kenu, Samuel Sackey, Kennedy Kwasi Addo, Edwin Afari, Dixon Chibanda, Bassirou Bonfoh

**Affiliations:** 1 University of Ghana, Accra, Ghana; 2 Ghana Health Service, Ministry of Health, Accra, Ghana; 3 Nelson Mandela African Institution of Science and Technology, Arusha, Tanzania; 4 Centre Suisse de Recherches Scientifiques en Côte d’Ivoire, Abidjan, Côte d’Ivoire; 5 Noguchi Memorial Institute of Medical Research, Accra, Ghana; 6 University of Zimbabwe, Harare, Zimbabwe; 7 Swiss Tropical and Public Health Institute, Basel, Switzerland; 8 University of Basel, Basel, Switzerland

**Keywords:** Livestock loss, Mental health, Veterinary, Food safety, Food security, Ghana

## Abstract

**Background:**

Agriculture represents the mainstay of African economies and livestock products are essential to the human population’s nutritional needs. However, in many developing countries, including Ghana, livestock production fails to meet demand due to population growth and negative effects of climate change. One of the challenges to production is livestock loss affecting farmers. However, despite stressful events experienced, livestock farmers’ mental health is poorly documented. This study aims to identify the root causes of livestock losses and their influence on pastoralists’ mental health.

**Methods:**

We conducted a mixed methods study in two districts in the Northern and Southern Belts of Ghana. Using the Depression Anxiety and Stress Scale–21 and guided interviews, we collected quantitative and qualitative data from 287 livestock farmers and 24 key-informants respectively. Mental health scores were categorized using standard guidelines. We evaluated the factors that explained variations in mental wellbeing using general linear models (*α* = 0.05).

**Results:**

About 85% (240/287) of the livestock farmers lost cattle within 1 year. Of these, 91% lost cattle to animal diseases, 50% to theft and 27% to pasture shortages. Qualitative findings reveal that due to poor access to veterinary services, farmers treat livestock diseases themselves with drugs from unregulated sources and often sell diseased cows for meat to recover losses. Findings showed that 60% of livestock farmers had poor mental health. Of those, 72% were depressed, 66% anxious and 59% stressed. Mental wellbeing was negatively associated with the number of adverse events experienced, proportion of livestock lost to most of the major loss factors, emotional attachment to livestock and self-reported physical illnesses in farmers, but positively associated with increasing herd size [F (8,278) = 14.18, *p* < 0.001, *R*^*2*^ = 0.29].

**Conclusions:**

Livestock diseases are the leading cause of losses to livestock farmers, whose mental wellbeing is negatively affected by these losses. Although an adaptive strategy by farmers to compensate for poor veterinary services, the arbitrary use of veterinary drugs and sale of diseased cattle pose health risks to the public. Further research to evaluate the performance of veterinary services in Ghana, mental health problems and risk to human health due to potential high-risk meat entering the food chain, is needed.

## Background

Agricultural activities represent the main source of livelihood for a vast majority of the poor in developing countries, where it is mainly operated on a subsistence basis and contributes to the local and global economy through trade. Livestock production is a big part of this and animal products serve as a key protein source in the human diet [1]. In spite of agriculture’s contribution to the economies of developing countries, it is linked to different forms of adverse events [2] which are a source for psychosocial problems to farmers. Mental problems arise when people are confronted by adverse events that are left unaddressed, affecting their normal day-to-day functioning.

Livestock farmers in Africa face multiple adverse events, all of which have increased in incidence recently [1], including disease outbreaks, drought, and conflict. The increased incidence of adverse event has been attributed to climate change [3]. Due to this, farmers often lose livestock and are left in a traumatic situation [4]. According to the International Labour Organization (ILO), occupational pressures in any workplace represent the leading source of psychological problems in adults, with high stress levels at work negatively affecting productivity [5]. The mental health of individuals is therefore a key predictor of their productivity. In a farming context, the loss of livestock, thus, affect the production levels of livestock farmers in the midst of increasing demand from a growing population, creating a mismatch between the demand for livestock products and what is actually being produced [6].

Even though mental health problems are one of the recognized non-communicable diseases with high burden in both developed and developing countries, it has received less focused attention over the past decades, especially in a rural context [7]. The World Health Organization (WHO) estimates that each year, more than 450 million people across the general population suffer from mental illness globally and about 75% of people with mental disorders in developing countries, where farming is the only source of income for many, receive no treatment [8]. Additionally, mental disorder-related deaths occur mainly through suicides, accounting for about 1 million deaths a year globally [9]. In Ghana, an estimated 3 million persons were living with mental disorders in 2007, with a treatment gap of more than 95% [10]. A nationally representative survey conducted in Ghana between 2009 and 2010 also found that about 20% of the general population have psychological distress [11]. The government of Ghana passed the Mental Health Act 2012 (Act 846) in the same year, with the goal of improving the mental healthcare delivery in the country [12].

Against this background, the United Nations’ Sustainable Development Goals (SDGs) One (1) through Three (3) were formulated, to improve health outcomes, including mental health, food security, and to reduce poverty globally. In spite of the strategies implemented to attain the set indicators of these goals, the United Nations Department of Economic and Social Affairs reports slow progress of some of the indicators in Africa and proposed a proper execution of agricultural practices as the key solution to self-sustainability on the continent [13].

Livestock production in Ghana has stagnated since 2009, and even fell from 32 to 22% in 2013 [14], even though over 40% of households in Ghana are involved in livestock rearing [15]. According to the estimates by the Ministry of Food and Agriculture (MoFA), cattle production in Ghana increased by only 7.6% between 2000 and 2010 [16]. Over the same period, Ghana’s population grew by more than 30% [15]. The consequences of this mismatch in growth include high meat imports, high meat prices, and loss of foreign exchange. The production gap is reflected in the total beef imports into Ghana, that have been exponentially increasing by 1876.4%, from a little over 600 metric tons in the year 2000 to about 12,500 metric tons in 2010 [16]. The global demand for livestock products is estimated by the Food and Agriculture Organization to further double by 2050 [6]. Therefore, there is the urgent need to identify the causes for loss of livestock and mental health problems in farmers to increase production and wellbeing in order to inform appropriate interventions to address these. Food insecurity in Ghana will increase substantially, if livestock production levels in Ghana are not improved. Identifying the root causes of livestock losses and the drivers of farmers’ mental health are key to meeting the SDGs 1, 2 and 3.

## Methods

### Description of study areas

**Fig. 1:**
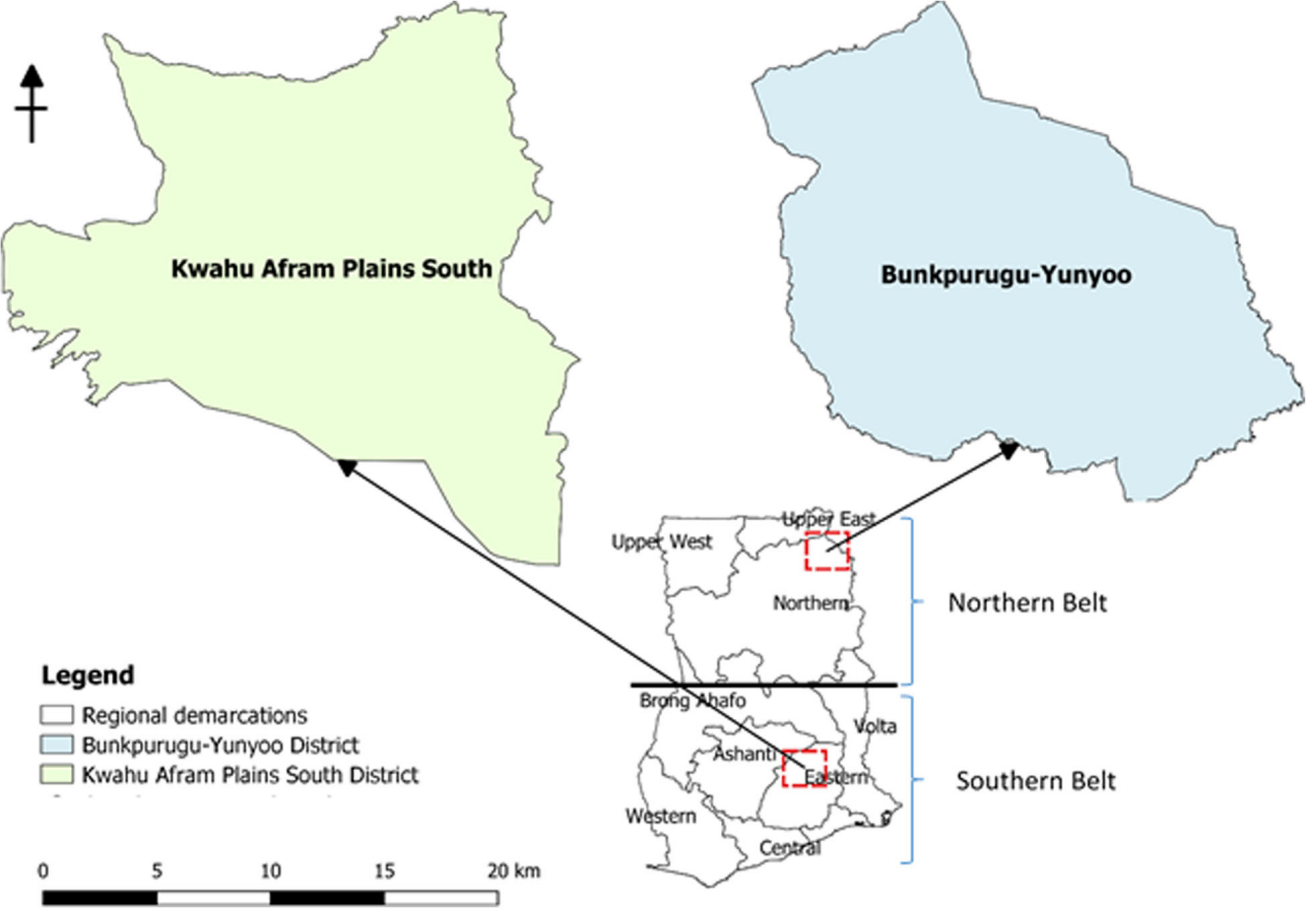
Map of Ghana showing the Bunkpurugu-Yunyoo (BY) and Kwahu Afram Plains South (KAPS) Districts. Map created by the authors using the Quantum Geographic Information Systems (QGIS) Software version 3.6

This study was carried out in two representative districts of the Northern and Southern Belts in Ghana. The Bunkpurugu-Yunyoo (BY) District in the Northern region of Ghana and Kwahu Afram Plains South (KAPS) District in the Eastern region were drawn randomly from a list of 14 districts in the Northern Belt and seven in the South, of the highest livestock production districts respectively (Fig. 1).

The BY District is an agrarian settlement which lies within the Northern Savannah Agro-ecological zone and has common grass vegetation. The landscape is generally gently rolling and the climatic conditions make the district conducive for the cultivation of food and cash crops, and the rearing of livestock. More than 94% of households within the district are engaged in agricultural activities. There is only a single rainfall season annually, usually between April and October with a mean annual rainfall ranging between 100 mm to 115 mm. The traditional authorities allocate land portions to persons who wish to engage in agriculture. Livestock and poultry production is mostly done under a free-range system with animals including chickens, goats, sheep, cattle, and pigs reared by farmers [17].

The KAPS District is also an agricultural settlement with over 80% of the population engaged in agricultural activities including crop production, livestock rearing, and fish farming. The landscape is generally undulating with vast stretches of arable land that lies within the Savannah vegetation zone. The district has the Volta River, which flows in the East into the Gulf of Guinea. The soils are suitable for the cultivation of both food and cash crops. There are two main rainfall seasons; the first rainy season starts from May to June and the second from September to October. The mean annual rainfall is between 1150 mm and 1650 mm. Livestock rearing is the second most important agricultural activity in the district after crop farming and is operated mainly on a free-range basis. The main animals reared in the district are cattle, goats, and sheep. The Kwahu Traditional Council, the highest traditional authority in the district, allocates land portions to persons interested in farming [18].

### Research design

This was a cross-sectional survey using a concurrent mixed-method approach. Multistage sampling was adopted in selecting the livestock farmers for the quantitative study. To ensure comparability of the value of losses suffered, only livestock farmers rearing cattle were recruited. Firstly, we identified and stratified cattle farming communities into the Northern Belt (farming in the Northern Savannah zone) and Southern Belt (farming in the Savannah vegetation zone). Accordingly, 55 and 40 cattle farming communities, from the BY and KAPS Districts respectively, were included from which 12 communities in the BY District and 10 communities in the KAPS District were randomly selected (See Additional file 1). From the selected communities, 145 and 142 cattle farmers in the BY and KAPS districts respectively were recruited consecutively from a sampling frame provided by community leaders of the farmers. For the qualitative approach, 19 farmers’ leaders in the communities were selected purposively; ten (10) from BY and nine (9) from KAPS. In addition, all five (5) veterinary officers (two (2) from BY and three (3) from KAPS), were recruited into the qualitative study. Overall, 22 communities were sampled from the two districts. A total of 287 cattle farmers were selected for interviews with a structured questionnaire, while nineteen (19) cattle farmers’ leaders and five (5) veterinary officers took part in in-depth interviews. Factors that were assessed were based on literature review of previous studies.

### Data collection and analysis

The research team comprising the principal investigator and three research assistants visited the cattle farmers and veterinary officers in their homes and/or workplaces to administer the questionnaires between 1st July and 29th September 2018. Two questionnaires; structured and unstructured, were administered to the respondents face-to-face, in one of four (4) languages, English, Ewe, Akan or Bimoba, depending on their language preference, to obtain quantitative and qualitative information respectively. The Depression, Anxiety and Stress Scale – 21 (DASS-21) was used to assess the mental health of the cattle farmers. It was not possible to do a full-scale validation of the DASS-21 for our study population [19]. Thus, two experts each in the three local languages carried out translation of the tools using the back translation approach [20]. Other quantitative data collected included sociodemographic characteristics, sources of losses and number of cattle lost, emotional attachment to livestock and support available to the farmers. To complement this quantitative data, 19 leaders from the sampled communities and 5 veterinary officers were interviewed as key informants.

The in-depth interviews were conducted using separate interview guides for the leaders of cattle farmers and veterinary officers in both study districts. The farmers’ leaders interviewed shared their experiences on what livestock rearing meant to the farmers, the key adverse events leading to livestock losses to the farmers, the coping strategies adopted, and the support available to farmers to deal with these events. The discussions with the veterinary officers captured their perspectives on the nature of services provided to the livestock farmers, common challenges faced in their work, and the utilization of veterinary services by the farmers.

The quantitative data collected was coded and analyzed using STATA software (version 15.1) [21]. Descriptive analysis of the data was expressed as frequencies and proportions for categorical data, and means with standard deviations for continuous data. The DASS – 21 Likert scale scores ranging between 0 and 3, 0 meaning non-experience of the negative emotion and 3 as frequent negative emotion experience, were reversed so that higher scores denote better mental health; 0 = 3, 1 = 2, 2 = 1 and 3 = 0. The reversed scores were summed under each of the three subscales (depression, stress, and anxiety), and multiplied by 2 to generate depression, stress and anxiety scores. The mean of these three scores was computed to generate the mental health score. The mental health scores were categorized into two; scores above and below the mean mental health score depicting better and worse mental health respectively. Inferential analysis was done to identify the factors that explained variations in the mental health categories using Chi-square test. To determine the most efficient linear model of mental health and predictors, a backward elimination model selection was done using likelihood ratio tests, starting with the most plausibly complex model [22]. Six (6) stepwise regression models were run, starting with 13 independent variables in the first model. Likelihood ratio tests were used to eliminate the independent variables that were not statistically significant to obtain the most efficient predictor model with eight independent variables. In the multivariate linear analysis, the coefficient of determination (*R*^*2*^), F statistic, coefficients of the multiple linear regressions of each independent variable with their respective standard errors, were presented. Diagnostic analysis of the residuals of each regression model was performed.

The interview recordings from discussions with the leaders of livestock farmers and veterinary officers were transcribed. The transcripts were coded deductively using NVivo software (version 12) [23], to generate themes and sub-themes from the emerging patterns in the data relevant to the research questions. Triangulation of the findings was done to provide depth to the meaning of the quantitative results obtained. The findings were expressed as narratives supported by verbatim quotations (see Additional file 2 for the word cloud generated from qualitative analysis of the data).

## Results

### Farmers characteristics and causes of livestock losses

The mean age (SD) of the cattle farmers was 46.9 ± 11.7 years (range = 19 to 78 years). Almost all the respondents were male (93%) and had some basic education (46%). The average pastoral household had 10 members and almost all the respondents were married (92%). The majority of the farmers (70%) had experience with cattle rearing and raising livestock in general since their childhood. Household cattle holdings ranged between 11 and 400 herds (median = 31). The cattle farmers in the Kwahu Afram Plains South (KAPS) District had significantly larger herd sizes, with about 40% (51/142) having more than 50 cattle herds, compared with 21% (31/145) for those in the Bunkpurugu-Yunyoo (BY) District. More than 90% (261/287) of the cattle farmers grew crops including cereals (230/261), legumes (114/261), vegetables (49/261) and root tubers (39/261). One-third (90/287) of the respondents reported experiencing some illness at the time of the study. They reported symptoms including myalgia, asthma, skin lesions, eye problems and high blood pressure. Close to 50% (41/90) of the farmers reported musculoskeletal symptoms. About 80% (225/287) of the farmers were emotionally attached to their cattle. Farmers with larger than 50 cattle herds were more emotionally attached to their herds than those with smaller herds (less than 50), *p* = 0.033.

**Fig. 2:**
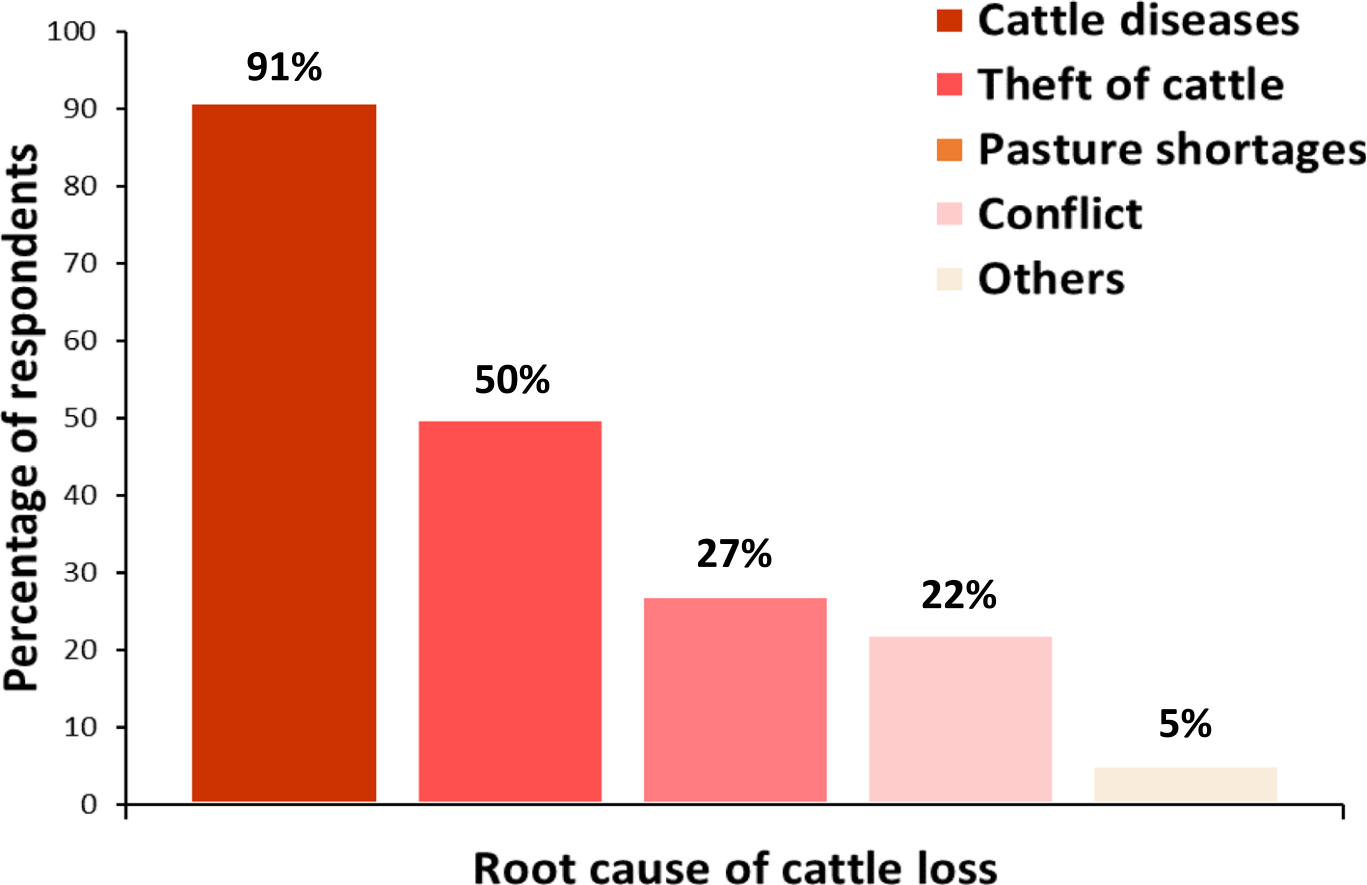
Root causes of livestock losses to farmers in Ghana (*n* = 240)

Around 85% (240/287) of the cattle farmers lost cattle over a one-year period. The median number of cattle lost per year per farmer was six (interquartile range = 3 to 10). Proportionately, the cattle farmers lost on average 15% of their total herd size within 1 year. The main adverse events accounting for the losses to 91% (218/240) of farmers were animal diseases. The other main risk factors for loss were from cattle theft (50%), pasture shortages (27%) and conflicts with other land users (22%) (Fig. 2). These losses have economic implications, costing each pastoralist on average about USD 1500, USD 600 and USD 300, to animal diseases, theft and pasture shortages respectively, per year (average value per head of cattle in Ghana = USD 300).

The findings from the qualitative enquiry indicate; the main diseases resulting in losses are Contagious Bovine Pleuropneumonia (CBPP), and Foot and Mouth Disease (FMD). CBPP is reported as the most severe cause of losses. These diseases were widely reported by both veterinary officers and the leaders of farmers in the study districts. As this farmer and veterinary officer remark:

*“For that heart disease (CBPP), I do not know. If it just enters your kraal, it takes time before it goes. It is not easy, not one year or two years. If you don’t take care, you will lose a lot. I know a farmer who had about 300 cattle, when this disease affected him. Before he realized, only 100 remained. Sometimes they go out to graze, and before you know it, five of them have died from that sickness; too much!”* (Male livestock farmer, 38 years, BY).

*“ … CBPP kills them a lot. Foot and mouth disease is not much of a problem because only the young ones die from it, because they are unable to suck with the sore in the mouth; for the CBPP, it is serious”* (Veterinary Field officer, 39 years, KAPS).

The majority of livestock farmers (170/287) received support from veterinary services to deal with adverse event occurrences. On average, the support interventions received by farmers per year come from two (interquartile range = 0 to 4) different of the following four main sources: veterinary services, friends and family or pastoralist association. The farmers with smaller herds (less than 50 cattle), reported receiving a significantly (*p* < 0.001) higher number of supporting interventions (median = 2, interquartile range = 0 to 4) compared to those with large cattle herds (median = 1, interquartile range = 0 to 2) (see Table 1). The farmers in the BY District also reported significantly higher average level of support available (median = 3, interquartile range = 1 to 4) compared to the farmers in the KAPS District (median = 1, interquartile range = 0 to 2) (*p* < 0.001).

**Table 1:** Sources of support available to livestock farmers to deal with adverse events (*N* = 287)

Source of support available	Small herd (< 50 cattle)	Large herd (≥50 cattle)	Total frequency (n)*
Veterinary services	119	51	170
Family	104	47	151
Friends	76	37	113
Pastoralist association	61	36	97
Insurance	4	4	8
Government compensation	1	1	2

*Frequencies denote multiple responses of each respondent for type of support interventions received in a year

The qualitative findings however, show that veterinary support to farmers is insufficient. The veterinary officers attribute this challenge to inadequate logistics (human and physical resources) and unwillingness of the cattle farmers to utilize vaccination services. Owing to this inadequate service delivery, the farmers resort to buying veterinary drugs from mainly unlicensed dealers and treat cattle diseases themselves. As these farmers’ leaders and veterinary officers indicate:

*“ … you see especially Bunkpurugu here, the farmers don’t want to vaccinate their animals. Because of that, we are facing many problems like PPR, CBPP, and anthrax … we see CBPP and PPR always. That one is not going at all”* (Veterinary Field officer, 27 years, BY).

*“Our animals die because of lack of attention from the veterinary officers. Veterinary services are very, very poor. You go to them and they say there is no medicine. So the veterinary services itself is not responding well to the livestock farmer. I cannot tell you the number of times that they even come to check on our animals. It is virtually nil”* (Male livestock farmer, 72 years, BY).

*“The challenge has to do with personnel. The area is so vast and we are seriously understaffed. The farmers live in areas that are so far apart, so at times, hmmm, it is difficult to get to all of them...We know there are some quacks going round, injecting the animals, but because we are under-staffed, I don’t fight with them*” (Doctor of Veterinary Medicine, 51 years, KAPS).

*“Sometimes the farmers do not even know how to administer the drugs properly. Everyone just does what they think is right. Some farmers buy medicine in a red and yellow bottle and mix with “Akpeteshie” before injecting the animals. So sometimes, I think we the farmers ourselves kill the animals because the injections are not given properly and we do not even know the right quantity to give. If the drugs are given in excess, the animal may die and people just smoke the meat and send to the market”* (Male livestock farmer, 47 years, KAPS).

As the last farmer indicated, selling of diseased cattle for meat on the market to recover losses is one of the adaptive strategies adopted by the livestock farmers to cope with the adverse events they suffer. The veterinary services are unable to regulate the drugs used by farmers in treating animals and to ensure that only wholesome livestock products enter the food chain:

*“For most of the farmers, they will sell the dead ones; they will not throw it away. If the animal dies, some of them will smoke the meat nicely and send it to market as bush meat. That is what they do”* (Veterinary Field Officer, 39 years, KAPS).

*“These diseases that affect our animals, pose a health threat to those in the cities. If the meat is unwholesome and the farmer doesn’t discard it, it will be sent there”* (Male livestock farmer, 47 years, KAPS).

*“We do not eat the cattle if it dies of diseases. So we have to sell at very low prices, sometimes 700 Ghanaian Cedis (USD 128). You lose a lot due to this”* (Male livestock farmer, 38 years, BY).

Despite the immense contribution of a lack of sufficient veterinary staff to decreased livestock farmers’ access to veterinary services, some of the impediments are purely financial. The veterinary officers report having to pre-finance the purchase of veterinary drugs and consumables they use in treating livestock and thereafter charge the livestock farmers for the services delivered.

*“Sometimes when you call the doctor, he will say his fuel, car, etc. cost him, and then charges you a higher amount that I cannot afford. That is how they are. Sometimes it is just a problem with one animal. For this, you will waste all that money?”* (Male livestock farmer, 49 years, BY).

*“ … somebody (farmer) will call you to come and treat the animals. Then you go to these places to vaccinate or treat and he will not give you the money. Another day, he will not call you. He will call another veterinarian, even though he owes you, he will just change the veterinarian … So the next time you call me, I will not go”* (Veterinary Field Officer, 27 years, BY)*.*

*“Sometimes they (farmers) agree with you to administer the drugs on credit. Thereafter, the payment becomes a problem...So, next time if you call me, I won’t bother to go and do the work for him, because it will affect me. I have taken money from my small income to buy the drugs to work for you and you do not want to pay me the money back?”* (Veterinary Field Officer, 39 years, KAPS)*.*

The study found that the majority of the cattle farmers (60%) have poor mental health. Of those, 72% were depressed, 66% anxious and 59% stressed. The mean mental health score was 45.7 (SD = 5.7) out of a possible 63. On average, the farmers scored 42.7 (SD = 7.4) on the stress scale, 47.5 (SD = 6.4) on the anxiety scale and 46.8 (SD = 6.6) on the depression scale out of a possible 56 for each of the sub-scales. The livestock farmers performed better on the anxiety sub-scale of the mental health assessment. Even though the average mental health levels were not statistically different between the study districts, the livestock farmers in the KAPS District had relatively better mental health than the livestock farmers in the BY District (see additional file 3).

### Causes of livestock farmers’ poor mental health

The mental health of the cattle farmers is influenced significantly by the age of the livestock farmer, the farmers’ emotional attachment to their livestock, the number of stressful events experienced and the proportion of livestock lost as well as the farmers’ health status (Table 2).

**Table 2:** Factors influencing the mental health of livestock farmers

Factor	Category	Poor (*n* = 171)	Good (*n* = 116)	Percent (%)	Statistical significance	
					Chi-square	***P***-value
Age of farmer	Less than 50 years	90	81	59.6	8.487	0.004
	50 years and above	81	35	40.4		
Sex	Female	15	4	6.6	3.169	0.092^a^
	Male	156	112	93.4		
Highest level of education	No formal education	56	40	33.4	1.782	0.642^a^
	Basic	81	51	46.0		
	Secondary	24	21	15.7		
	Tertiary	10	4	4.9		
District	Kwahu Afram Plains	80	62	49.5	1.228	0.268
	South	91	54	50.5		
	Bunkpurugu-Yunyoo					
Marital status	Not married	12	10	7.7	0.251	0.616
	Married	159	106	92.3		
Number in household	10 or fewer members	93	73	57.8	2.070	0.150
	More than 10 members	78	43	42.2		
Number of cattle in herd	50 or fewer cattle	117	88	71.4	1.875	0.171
	More than 50 cattle	54	28	28.6		
Cultivation of crops	No	15	11	9.1	0.042	0.837
	Yes	156	105	90.9		
Raised with farm animals	No	45	42	30.3	3.201	0.074
	Yes	126	74	69.7		
Support availability	No support (≤2 sources)	109	70	62.4	0.340	0.560
	Support (> 2 sources)	62	46	37.6		
Level of attachment	Not attached	24	38	21.6	14.307	< 0.001
	Attached	147	78	78.4		
Self-reported illness status	Not ill	103	94	68.6	13.892	< 0.001
	Ill	68	22	31.4		
Level of livestock loss	No loss (0%)	16	31	16.4	19.556	< 0.001
	Low loss (< 10%)	49	32	28.2		
	Moderate loss (10–25%)	68	42	38.3		
	Severe loss (> 25%)	38	11	17.1		

Numbers (n) of farmers falling into each category of ‘good’ and ‘poor’ mental health, percentage (%) denotes the proportion of farmers with poor mental health within each influencing factor category and their Chi-square and *p*-value. ^a^denotes Fisher’s exact test probabilities for observations less than 5 persons in each mental health category. Poor and good mental health categories denotes scores of farmers less than and above the study average of 45.7 respectively

Table 3 below illustrates the model selection iteration to determine the most efficient linear model predicting the mental health of cattle farmers. The best model had eight independent variables and accounted for about 30% of the variance in mental health (Model 6). After controlling for the farmers’ marital status, number of support received, district of farming, growing of crops and farmers’ experience with raising livestock, the cattle farmers’ mental wellbeing was negatively influenced by the number of adverse events experienced, report of ill health by the farmers, farmers’ emotional attachment to livestock, and proportion of cattle lost to diseases, theft and conflict. Whereas a unit increase in a farmers’ herd size and losses suffered from pasture shortages, were associated with an improvement in their mental wellbeing [*F* (8, 278) = 14.18, *p* < 0.001, *R*^*2*^ = 0.29] (Table 3). It is also important to note that elimination of other variables in the subsequent models did not result in significant changes in the strength of the relationship (slope) and the statistical significance of the individual predictor variables compared to Model 1. We also present simple general linear models demonstrating the effects of the proportions of livestock lost to the major factors including animal diseases, theft, pasture shortages, conflicts and the total proportions of herd lost to all causes on mental health of livestock farmers in additional information 4.

**Table 3:** Backward elimination regression analysis for factors predicting farmers’ mental health (*n* = 287)

	Model 1	Model 2	Model 3	Model 4	Model 5	Model 6
**Variable**	Coef. (*SE* β)	Coef. (*SE* β)	Coef. (*SE* β)	Coef. (*SE* β)	Coef. (*SE* β)	Coef. (*SE* β)
Self-reported ill health status	−2.91 (0.68)^d^	−2.92 (0.68)^d^	−2.93 (0.66)^d^	−2.93 (0.66)^d^	−2.92 (0.66)^d^	−2.96 (0.66)^d^
Number of adverse events	−0.76 (0.21)^d^	−0.76 (0.21)^d^	−0.76 (0.21)^d^	−0.76 (0.21)^d^	−0.81 (0.18)^d^	−0.83 (0.18)^d^
Proportion of herd lost	−0.07 (0.02)^c^	− 0.07 (0.02)^c^	− 0.07 (0.02)^c^	− 0.07 (0.02)^c^	− 0.07 (0.02)^c^	− 0.07 (0.02)^c^
Emotional attachment to cattle	−1.86 (0.77)^b^	−1.86 (0.77)^b^	−1.82 (0.77)^b^	−1.82 (0.77)^b^	−1.80 (0.76)^b^	−1.82 (0.76)^b^
Herd size	0.02 (0.01)^a^	0.02 (0.01)^a^	0.02 (0.01)^b^	0.02 (0.01)^b^	0.02 (0.01)^b^	0.01 (0.01)^b^
Experience with livestock	−0.72 (0.67)	−0.72 (0.66)	− 0.72 (0.66)	−0.72 (0.66)	− 0.73 (0.66)	
District (Bunkpurugu)	−0.48 (0.76)	−0.48 (0.73)	− 0.37 (0.70)	−0.37 (0.70)		
Cultivator of crops	0.61 (1.11)	0.62 (1.10)	0.61 (1.10)			
Household size	0.01 (0.06)	0.01 (0.06)				
Number of support sources	0.01 (0.21)					
*Slope*	54.52	54.52	54.58	54.99	54.70	54.44
*R*^*2*^	0.24	0.24	0.24	0.24	0.22	0.23
*F-ratio*	8.68^d^	9.68^d^	10.92^d^	12.47^d^	14.54^d^	17.19^d^

“Coef” denotes coefficients that are unstandardized regression slopes with standard errors (SE β) in parentheses. ^a, b, c, d^, denote 10, 5, 1 and 0.1% significance level respectively. “Variable” = Variables included as predictors of the mental health of livestock farmers in Ghana. Categorical variables and their levels, added in the regression include self-reported ill health status (0 = Not ill/1 = Ill), Emotional attachment to cattle (0 = Not attached/1 = Attached), has experience raising livestock since childhood (0 = No/1 = Yes), District (0 = Kwahu Afram Plains South/1 = Bunkpurugu-Yunyoo), cultivator of crops (0 = No/ 1 = Yes). All ‘0’ denote the reference category. R2 is the coefficient of determination for each of the multivariate linear regression models

## Discussion

Agriculture and livestock production particularly contribute significantly to the growth and development of many developing countries including Ghana, and serve as a livelihood source to a vast majority of the poor in society. Despite these benefits, it is also one of the most stressful occupations. Due to negative effects of climate change, adverse events including droughts and conflict have increased in occurrence, leading to livestock losses to farmers and thereby affecting their productivity. Due to this decline in production of livestock, the local demand for livestock and livestock products is not adequately met, leading to high meat imports, high food prices and loss of foreign exchange. The loss of livestock may also lead to negative livelihood and mental health consequences for livestock farmers. Therefore, identifying the drivers of low mental health of livestock farmers and sources of livestock losses is key to inform interventions and being able to meet the Sustainable Development Goals (SDGs) 1, 2 and 3, which aim to reduce poverty, reduce hunger and ensure good health and wellbeing.

Our study provides evidence, that the root causes of livestock losses are from livestock disease outbreaks, cattle theft, pasture shortages and conflicts. The extent to which climate change affected the prevalence of these adverse events, though previously documented [3, 24, 25], could not be ascertained in this study. Livestock diseases were the leading cause of losses suffered by farmers because of mainly inadequate access to veterinary services and a lack of clarity on the responsibility of farmers, resulting in them treating animal diseases themselves using drugs freely available on the market, arbitrarily without veterinary prescription. These findings agree with a previous study conducted among livestock farmers in the Northern Region of Ghana [26]. Periodic education provided by veterinary extension workers to livestock farmers could address the problem of pasture shortages. The establishment of a fodder bank for storage of feeds against drought periods and provision of other veterinary services in Wawase in the Kwahu Afram Plains South District by the Ministry of Food and Agriculture in collaboration with the Africa Development Bank [27] is commendable. Cattle rustling in pastoral communities could be addressed if law enforcement is strengthened in farming communities in Ghana. Some of the farmers in the present study also sell diseased animals on the market to recover some losses. These practices appear to be a coping strategy adopted by the livestock farmers to help them deal with the challenge of inadequate access to veterinary services and thus high losses and is consistent with other studies [28, 29].

The loss of livestock was found to negatively affect the livelihood and reported physical and mental health of the livestock farmers. The farmers in this study lost an average of USD 1500 per year due to livestock disease outbreaks. This affected their ability to provide for the basic needs of their households. As expected, and as found in previous studies in the United Kingdom [30] and Australia [31], following the outbreak of Foot and Mouth Disease (FMD), livestock losses negatively affect the mental health of farmers. The proportion of the livestock farmers with psychological distress (60%) is relatively high, compared to the previously reported prevalence of psychological distress of 20% among the general Ghanaian population [11]. This finding is not uncommon, as other comparative studies in Europe, America and Australia showed agricultural workers to be highly stressed compared to other occupations [32–34]. The main diseases leading to the losses in Ghana include FMD and Contagious Bovine Pleuropneumonia (CBPP). The occurrence of disease outbreaks of CBPP and FMD were widespread in both study districts. This is despite the availability of vaccination against FMD. It is surprising though that the losses suffered by the farmers to pasture shortages appear to be positively related to mental health with each loss. This may be possible if the farmers attribute losses to normal seasonality of the weather and may be expectant of better conditions during different times of the year.

The contrasting accounts given by the livestock farmers and veterinary officers alike on the reasons for the high prevalence of livestock diseases is worth exploring. While the livestock farmers complained of a delayed or absent response by the veterinary officers to requests for veterinary services, the veterinary officers on the other hand, while acknowledging staff challenges, report that the livestock farmers do not patronize preventive services, especially their vaccination services when organized. The lack of and/or poor uptake of veterinary services appears to be mostly financial in nature. As findings showed, the veterinary personnel often have to pre-finance the drugs and the consumables used in administering them. Thus, they often have to balance their quest to recuperate their investments with the livestock farmers’ need for veterinary services. These facts point to a lack of clearly defined roles and responsibilities for livestock farmers, veterinary officers and the government in the livestock production system. Farmers had taken up duties of veterinarians by administering drugs including intravenous medications, while veterinary officers have become private entrepreneurs in a public service sector. What is clear though is the insufficient level of veterinary services support to livestock farmers.

In addition, as qualitative findings revealed, most livestock farmers do not utilize vaccination services organized, but prefer treating the diseases instead. However, some farmers also report a lack of vaccines at veterinary offices when required. In the instances where vaccinations are organized, the veterinary officers operate with an expectation that farmers would pay them back. This arrangement is problematic, as it may result in neglect of farmers by veterinary officers who do not have adequate finances of their own, when the Ministry of Food and Agriculture fail to own this crucial aspect of veterinary service delivery. The inadequate number of veterinary personnel and other logistical constraints undermine the efforts of the ministry to improve livestock production.

Consequently, the livestock losses to animal diseases have been enormous with economic and health consequences. The livestock farmers in Ghana would benefit immensely from awareness campaigns on preventive services like vaccination of livestock, to reduce economic loss, grief from cattle losses and improve cattle productivity. A similar intervention study among smallholder cattle farmers in Cambodia showed incremental gains in cattle weights, improved farmer knowledge levels and had a positive impact on household income over time for smallholder farmers [35]. While the economic influence of livestock losses in this study was evident, many of the livestock farmers have also developed a special bond with their livestock. Livestock rearing, therefore, is more than just a source of livelihood to the farmers. These findings are consistent with qualitative inquiries conducted in Maasai communities in Eastern Africa, in Australia and the Alps of Switzerland which found deep emotional bonds between livestock farmers and their cattle [36–38]. This emotional attachment could emanate from the close contact the livestock farmers have with the cattle during milking, feeding, and cleaning of pens among others, which are practices similar to the earlier studies and this present one. The livestock farmers manifest their bond to cattle by talking to the cattle, giving names to cattle and caring for their furs. The emotional attachment to livestock tended to amplify the negative effect of loss on the mental health of farmers, as our findings revealed. The stronger bond of farmers with larger herds is surprising, but may explain why more farmers with large herds tend to seek veterinary care for their sick animals compared to those with smaller herds; apart from the obvious fact that farmers with larger herds are more likely to have resources to pay for veterinary services.

The state of mental health of livestock farmers in Ghana is worrying. While we found in this study that about two-thirds of livestock farmers had psychological problems, previous studies in Ghana showed a prevalence of psychological distress in less than one-third of the general population [11]. This finding is similar to previous research by the International Labour Organization which identified agriculture as one of the most stressful occupations [32]. The high levels of stress livestock farmers are confronted with from multiple adverse events they suffer could be contributory to the higher than normal prevalence of psychological problems among farmers compared to the general population. Additionally, the tendency of livestock farmers to live in areas isolated from human settlements in order to gain access to pasture as well as avoid confrontations with other land users, limit their access to health services that are not readily available in rural areas in developing countries. The Mental Health Authority in Ghana would be instrumental in devising strategies to make mental health services more accessible to farmers. The state of a farmers’ physical health also affected their mental health. The livestock farmers’ that reported some physical ill health were more likely to have psychological problems compared to those without physical ailments. Improved access to health services can therefore greatly influence the general health of livestock farmers.

Nevertheless, the ability of the livestock farmers to adapt easily to the lack of veterinary services by learning to treat diseases themselves and selling of diseased meat can be described as a resilience strategy. This is one of many other adaptive strategies of livestock farmers to adverse events found in other studies conducted in East Africa [39, 40]. This presents an opportunity that can be harnessed by the appropriate government agencies as farmers show a willingness to do everything possible to keep their herd healthy. The timely provision of support services to farmers can therefore contribute to increased productivity and attaining food security and general growth and development. The recently launched program, “rearing for food and jobs”, by the Ministry of Food and Agriculture in Ghana, will come to nothing if the shortfalls in veterinary service delivery are left unaddressed.

More so, the arbitrary use of freely available veterinary drugs to treat diseases and the sale of diseased livestock for meat on the markets by livestock farmers, presents potential food safety concerns. The insufficient number of veterinary personnel would mean that not all meat presented at the markets would have been cleared to be wholesome for the consumption of the public. This is also a concern for the emergence of antimicrobial resistance (AMR) in retailed food. The identification of AMR bacteria including: *Campylobacter, Enterococcus, Salmonella, Escherichia coli, Listeria,* and *Vibrio spp*., in meat have been attributed to the indiscriminate use of veterinary drugs by farmers to treat animal diseases [41–43]. Hence, the formulation of the AMR policy by the government of Ghana through the Ministries of Food and Agriculture, Health, and Environment, Science and Technology is commendable. The policy is very well formulated, but requires concerted efforts to implement the strategies outlined.

We have demonstrated how the health of humans, animals and the environment depend on each other. There is a need for sensitization campaigns for the key stakeholders in the livestock production system. These interventions can increase vaccination rates and availability of vaccines supplied by the government, and forestall the negative practices. It is also essential that an evaluation of the performance of veterinary services in Ghana is conducted. Considering the potential of transferring resistant bacterial strains from food to humans, there is need for an intensified surveillance by veterinary personnel at livestock markets and slaughter areas to reduce the sale of veterinary drugs and diseased meat. We propose a future study, which would assess for resistant pathogens and drug residues in retail livestock products and explore any links with resistant pathogens which are isolated from human patients presenting with foodborne illnesses in Ghana though the utilization of the One Health approach to explore ways of maximizing the benefits accruable from the implementation of these policies.

Our study revealed concerning findings, by showing the complexity of the challenges faced by livestock farmers in Ghana. Inadequate veterinary services support together with non-adoption of preventive measures such as vaccinations, increases the frequency of adverse events resulting in livestock loss, and poor mental health. The prevalence of psychological problems among farmers is higher than observed in the general population in Ghana. What is more alarming, the coping strategies employed by farmers include arbitrary drug use for treating animal diseases and the sale of unsafe meat at local markets. These findings raise concerns about the low quality of life of livestock farmers and threats to public health through high-risk meat entering the food chain.

## Conclusions

The livestock farmers in Ghana face multiple stressful events, such as animal disease outbreaks, cattle theft and pasture shortages as well as conflicts with other land users, all of which lead to livestock losses. The loss of livestock, mainly due to a lack of sufficient veterinary support to farmers, negatively affects the productivity of the farmers, with livelihood and mental health consequences. The livestock farmers have adapted to this shortfall in veterinary support; treat animal diseases themselves and sell diseased meat in the markets, posing health risks to the public. The mismatch between the farmers’ needs and the veterinary services provision has to be addressed to improve mental health, productivity and reduce the threats to food security and safety.

## Supplementary information

**Additional file 1.** Sampling frame of livestock farming communities in Bunkpurugu-Yunyoo (BY) and Kwahu Afram Plains South (KAPS) Districts. This is a list of farming communities from which the 12 and 10 communities in the BY and KAPS Districts respectively, were randomly selected for the study.

**Additional file 2.** Word cloud showing key issues of concern to study participants. This is a figure generated from transcripts of interviews of key informants using NVivo software, showing the key concerns raised by the livestock farmers.

**Additional file 3.** Mental health scores of the livestock farmers by study district. This is a figure (box plot) of mental health scores of the study respondents by district of farming.

**Additional file 4. **Summary of simple regression analyses the effect of loss factors on livestock farmers’ mental health (*N* = 287). This is a table showing the individual effect of each loss factor on farmers’ mental health.

## Data Availability

All data generated or analyzed during this study are included in this published article and its supplementary information files.

## Abbreviations

AMR: Antimicrobial resistance
BY: Bunkpurugu-Yunyoo
CBPP: Contagious Bovine Pleuropneumonia
DASS-21: Depression, Anxiety and Stress Scale – 21
FMD: Foot and Mouth Disease
ILO: International Labour Organization
KAPS: Kwahu Afram Plains South
SDGs: Sustainable Development Goals
WHO: World Health Organization

## References

[1] FAO, IFAD, UNICEF, WFP, WHO (2018). The State of Food Security and Nutrition in the World 2018. Building climate resilience for food security and nutrition.

[2] Padhy C (2018). Stress among farmers and its alleviation. Int J Manag Technol Eng.

[3] Tseren B. Reducing herders’ vulnerability to climate change through community-based pastureland management in Mongolia. Solut J. 2018;9(4) Available from: https://www.thesolutionsjournal.com/author/battsetseg-tseren/.

[4] Kolstrup CL, Kallioniemi M, Lundqvist P, Kymäläinen H, Stallones L (2013). International perspectives on psychosocial working conditions, mental health, and stress of dairy farm operators. J Agromed.

[5] International Labour Organization (2016). Workplace Stress: a collective challenge. World Day for Safety and Health at Work.

[6] FAO (2017). The future of food and agriculture: trends and challenges.

[7] WHO. Global burden of mental disorders and the need for a comprehensive, coordinated response from health and social sectors at the country level Report by the Secretariat. Geneva: WHO; 2011.

[8] WHO (2012). Making a difference in the lives of people with mental disorders.

[9] WHO (2018). Suicide: key facts.

[10] WHO. Mental health improvement for nations development: the country summary series, Ghana, vol. 7. Geneva: WHO; 2007.

[11] Sipsma H, Ofori-atta A, Canavan M, Osei-akoto I, Udry C, Bradley EH (2013). Poor mental health in Ghana : who is at risk ?. BMC Public Health.

[12] Walker GH, Osei A (2017). Mental health law in Ghana. BJ Psych Int.

[13] DESA. The sustainable development goals report 2016. NNew York: UN; 2016.

[14] FAO (2015). Socio-economic context and role of agriculture.

[15] Ghana Statistical Service. 2010 Population & Housing Census - summary report of final results. Accra: GSS; 2012.

[16] MoFA. Agriculture in Ghana, facts and figures (2010). Accra: MoFA; 2011.

[17] Ghana Statistical Service. District analytical report: Bunkpurugu yunyoo district. Accra: GSS; 2014.

[18] Ghana Statistical Service. District analytical report: Kwahu afram plains south. Accra: GSS; 2014.

[19] Oei TPS, Sawang S, Goh YW, Mukhtar F (2013). Using the depression anxiety stress scale 21 (DASS-21) across cultures. Int J Psychol.

[20] Tyupa S (2011). A theoretical framework for back-translation as a quality assessment tool. New Voices Transl Stud.

[21] StataCorp (2017). Stata Statistical Software: Release 15.

[22] Heinze G, Wallisch C, Dunkler D (2018). Variable selection – a review and recommendations for the practicing statistician. Biom J.

[23] QSR International (2018). NVivo qualitative data analysis software.

[24] Bett B, Jost C, Allport R, Mariner J (2009). Using participatory epidemiological techniques to estimate the relative incidence and impact on livelihoods of livestock diseases amongst nomadic pastoralists in Turkana South District, Kenya. Prev Vet Med.

[25] Mbyuzi AO, Komba EVG, Kimera SI, Kambarage DM (2014). Sero-prevalence and associated risk factors of peste des petits ruminants and contagious caprine pleuro-pneumonia in goats and sheep in the southern zone of Tanzania. Prev Vet Med.

[26] Alicic B, Ennali Y, Toric A (2014). Animal health services in rural Ghana.

[27] MOFA (2019). Afram Plains District Agric Development Project.

[28] Agonafir CN (2018). Pastoralist resilience: the roles of customary institutions in Central Afghanistan. Solut J.

[29] Mekuyie M, Jordaan A, Melka Y (2018). Understanding resilience of pastoralists to climate change and variability in the Southern Afar Region, Ethiopia. Clim Risk Manag.

[30] Mort M, Convery I, Baxter J, Bailey C (2005). Psychosocial effects of the 2001 UK foot and mouth disease epidemic in a rural population: qualitative diary based study. BMJ.

[31] Peck DF, Grant S, Mcarthur W, Godden D (2002). Psychological impact of foot-and-mouth disease on farmers farmers. J Ment Health.

[32] ILO (2011). Safety and health in agriculture.

[33] Brew B, Inder K, Allen J, Thomas M, Kelly B (2016). The health and wellbeing of Australian farmers: a longitudinal cohort study. BMC Public Health.

[34] McIntosh W, Spies E, Stone D, Lokey C, Trudeau A-R, Bartholow B (2016). Suicide rates by occupational group — 17 states, 2012. Morb Mortal Wkly Rep.

[35] Young JR, Rast L, Suon S, Bush RD, Henry LA, Windsor PA (2013). The impact of best practice health and husbandry interventions on smallholder cattle productivity in southern Cambodia. Anim Prod Sci.

[36] Ellis NR, Albrecht GA (2017). Climate change threats to family farmers’ sense of place and mental wellbeing: a case study from the Western Australian Wheatbelt. Soc Sci Med.

[37] Hoffet F. Farm animals: between pets and livestock- a case study from a Swiss alpine farm. Uppsala: Swedish University of Agricultural Sciences; 2015.

[38] Macgregor H, Waldman L (2017). Views from many worlds: unsettling categories in interdisciplinary research on endemic zoonotic diseases. Philos Trans R Soc B, Biol Sci.

[39] Goldman MJ, Riosmena F (2013). Adaptive capacity in Tanzanian Maasailand: changing strategies to cope with drought in fragmented landscapes. Glob Environ Change.

[40] Opiyo F, Wasonga O, Nyangito M, Schilling J, Munang R (2015). Drought adaptation and coping strategies among the Turkana pastoralists of northern Kenya. Int J Disaster Risk Sci.

[41] Magouras I, Carmo LP, Stärk KDC, Schüpbach-Regula G (2017). Antimicrobial usage and resistance in livestock: where should we focus?. Front Vet Sci.

[42] Jans C, Sarno E, Collineau L, Meile L, Stärk KDC, Stephan R (2018). Consumer exposure to antimicrobial resistant bacteria from food at Swiss retail level. Front Microbiol.

[43] McEwen S, Fedorka-Cray P (2002). Antimicrobial use and resistance in animals. Clin Infect Dis.

