# Supplementary material for: Poor mental health of livestock farmers in Africa: a mixed methods case study from Ghana
Source: BMC Public Health. 2020 Jun 1;20:825. doi: 10.1186/s12889-020-08949-2 (PMC7268426; doi:10.1186/s12889-020-08949-2)
Supplement: Supplementary file 1 — Additional file 1. Sampling frame of livestock farming communities in Bunkpurugu-Yunyoo (BY) and Kwahu Afram Plains South (KAPS) Districts. This is a list of farming communities from which the 12 and 10 communities in the BY and KAPS Districts respectively, were randomly selected for the study. [file 12889_2020_8949_MOESM1_ESM.docx]

# Additional file 1: Sampling frame of livestock farming communities in BY and KAPS Districts

**SAMPLING FRAME OF LIVESTOCK FARMING COMMUNITIES IN KWAHU AFRAM PLAINS SOUTH (KAPS) DISTRICT**

1. ABOKYRE
2. AGYATA
3. AMANHYIA
4. AMEDZOFE
5. AMEYAW
6. ATAKORA
7. ATONSU
8. BATOR-KOFE
9. BONKPATA
10. DEDESO
11. DIM SAKABO
12. DOMEABRA
13. DOTORPONG
14. EKYE
15. FASO-KRACHI
16. FORIFORI
17. FOSU-TOMEFA
18. GADORKOPE
19. GAVORKOPE
20. GODODONUKOPE
21. GODOPE
22. GODZIKOFE
23. HLIHADZI
24. KOMLA-KWAO
25. KWAME DWAMENA
26. KWASI-ADAE
27. KWASIKUMAH
28. MAFIKOPE
29. MRANDAN
30. NEKPOKOPE
31. NYAFRED
32. ODOTOMU
33. ODUMASUA
34. OFFINSO
35. OGBODOKOPE
36. SUMSE
37. SEMENHYIA
38. TEASE
39. TRIBU
40. TWENFOUR FASO
41. WAWASE
42. XEDZODZOE-KOPE

**SAMPLING FRAME OF CATTLE FARMING COMMUNITIES IN BUNKPURUGU-YUNYOO (BY) DISTRICT**

1. BAMANGO
2. BILFACO POIGA
3. BINDE
4. BOATERIGU
5. BUMBONG
6. BUNBUNA
7. BUNKPURUGU ZONGO
8. CHINTULUNG
9. DAGBAN
10. DALOUR
11. GBANKONI
12. GBANKURUGU
13. GBETMUNPAK
14. GBINGBANI
15. GOMSUKA
16. GUAGBIANG
17. JAGOUK
18. JANDERI
19. JILIK
20. JIMBALE
21. KAMBA KONKOOK
22. KAUK
23. KINKANGU
24. KPAGYALA
25. KPANLORI
26. KPEKPALGBENI
27. KPEMALE
28. KPENTAUNGKUNKWADAN
29. KUNPAK TAMBING
30. MAJIA
31. MANGOR
32. NAABAUK
33. NABULIK
34. NAJONG
35. NAKPANDURI
36. NAMANGAI
37. NAMGBAM
38. NAMPOUTIBAUK
39. NANGOBIK
40. NANYIAR
41. NASUAN
42. PAGNATIK
43. PULI
44. SAKBOUK
45. SANJAAK
46. TABOKURUGU
47. TATARA
48. TIENKINLU
49. TINKPANG
50. TOJING
51. TUNA
52. TUSUGU
53. WAWA
54. YUNYOO
