# Supplementary figures and images for: Poor mental health of livestock farmers in Africa: a mixed methods case study from Ghana
Source: BMC Public Health. 2020 Jun 1;20:825. doi: 10.1186/s12889-020-08949-2 (PMC7268426; doi:10.1186/s12889-020-08949-2)

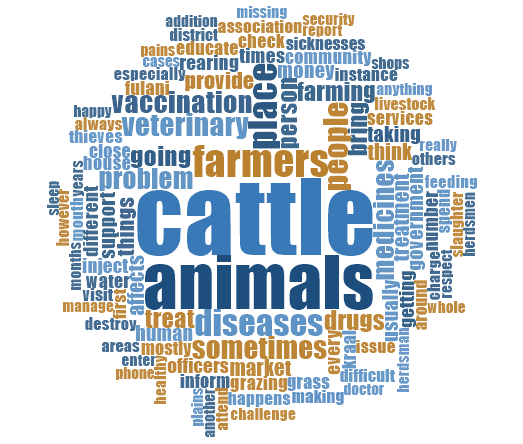


**Word cloud showing key issues of concern to study participants**

Supplement: Supplementary file 2 — Additional file 2. Word cloud showing key issues of concern to study participants. This is a figure generated from transcripts of interviews of key informants using NVivo software, showing the key concerns raised by the livestock farmers. [file 12889_2020_8949_MOESM2_ESM.docx]

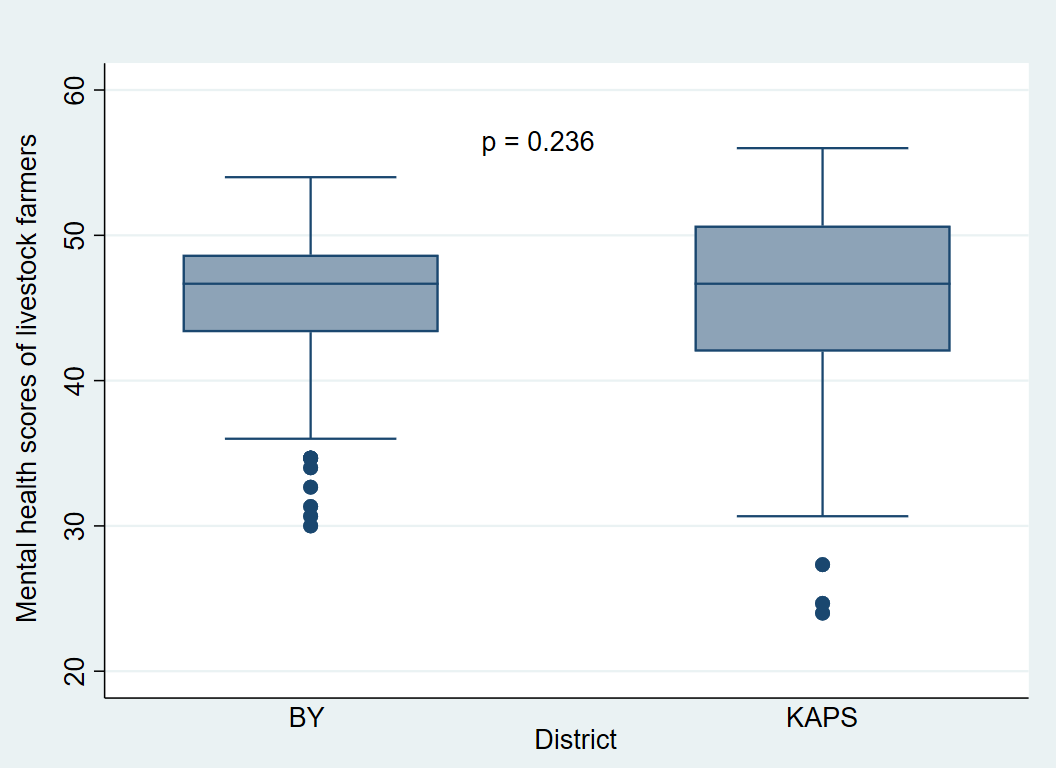


**Mental health scores of the livestock farmers by study district**

Supplement: Supplementary file 3 — Additional file 3. Mental health scores of the livestock farmers by study district. This is a figure (box plot) of mental health scores of the study respondents by district of farming. [file 12889_2020_8949_MOESM3_ESM.docx]
