# Supplementary material for: Poor mental health of livestock farmers in Africa: a mixed methods case study from Ghana
Source: BMC Public Health. 2020 Jun 1;20:825. doi: 10.1186/s12889-020-08949-2 (PMC7268426; doi:10.1186/s12889-020-08949-2)
Supplement: Supplementary file 4 — Additional file 4. Summary of simple regression analyses the effect of loss factors on livestock farmers’ mental health (N = 287). This is a table showing the individual effect of each loss factor on farmers’ mental health. [file 12889_2020_8949_MOESM4_ESM.docx]

**Summary of simple regression analyses the effect of loss factors on livestock farmers’ mental health (N = 287)**

|  | Wellbeing | | | |
| --- | --- | --- | --- | --- |
| Loss factor | *Slope* | *Standard error* | *t-statistic* | *p-value* |
| Animal disease | -0.08 | 0.03 | -2.33 | 0.021 |
| Theft | -0.21 | 0.06 | -3.80 | <0.001 |
| Pasture shortage | 0.27 | 0.14 | 1.94 | 0.054 |
| Conflict | -0.42 | 0.10 | -4.16 | <0.001 |
| Total proportion of cattle lost | -0.11 | 0.02 | -4.89 | <0.001 |
